# Peripartum depression symptom trajectories, telomere length and genotype, and adverse childhood experiences

**DOI:** 10.1186/s12888-024-06115-1

**Published:** 2024-10-08

**Authors:** Maria Vrettou, Susanne Lager, Simone Toffoletto, Stavros I. Iliadis, Theodora Kunovac Kallak, Sara Agnafors, Vanessa Nieratschker, Alkistis Skalkidou, Erika Comasco

**Affiliations:** 1 Department of Women’s and Children’s Health, Science for Life Laboratory, Uppsala University, Box 593, Uppsala, 751 24 Sweden; 2 Department of Women’s and Children’s Health, Uppsala University, Uppsala, Sweden; 3 Department of Biomedical and Clinical Sciences, Division of Children’s and Women’s Health, Linköping University, Linköping, Sweden; 4 Department of Psychiatry and Psychotherapy, Tübingen Center for Mental Health (TüCMH), Medical University Hospital Tübingen, German Center for Mental Health (DZPG), partner site Tübingen, Germany

**Keywords:** Telomere length, Peripartum depression, Adverse childhood experience, *TERT*

## Abstract

**Background:**

As a biological marker for cellular senescence, telomere length (TL) has been linked to a variety of psychiatric disorders and adverse childhood experiences (ACE), though only preliminarily to peripartum depression (PPD). The present study sought to examine the association between TL and PPD, assessing the moderating role of ACE and genetic polymorphic variations related with the telomere machinery.

**Methods:**

Adversity was self-reported, likewise were depressive symptoms evaluated at pregnancy week 17 and 32, as well as six-weeks and six-months postpartum. TL was assessed by use of qPCR in blood samples collected during delivery from females with antenatal depression resolving postpartum, females with depression persisting to postpartum, and healthy controls. Twenty haplotype-tagging Single Nucleotide Polymorphisms in the *Telomerase Reverse Transcriptase* (*TERT*) and three in the *Telomerase RNA Component* (*TERC*) genes were genotyped.

**Results:**

TL was negatively correlated with severity of PPD symptoms at pregnancy week 32 and postpartum week 6. PPD was associated with shorter TL. Lastly, ACE, but not the *TERT/TERC* genotype, moderated the TL-trajectory association; with increasing ACE, individuals with persistent PPD symptoms had shorter TL, whereas the opposite pattern (longer TL) was observed in the controls.

**Conclusions:**

The findings contribute to further understanding of PPD underpinnings, suggesting a negative relationship with TL.

**Supplementary Information:**

The online version contains supplementary material available at 10.1186/s12888-024-06115-1.

## Background

Over the last decade, the prevalence of depression and anxiety disorders has been increasing in young females in Sweden [1]. Peripartum depression (PPD) is occurring during pregnancy or within the first four weeks following delivery and its pathogenesis is likely multifactorial [2]. PPD affects around 12–20% of pregnant and postpartum females globally [3–5]. This constitutes a considerable personal and public health challenge [6]. Moreover, trajectories based on onset of symptom development in PPD point to possible PPD subgroups, such as antenatal depression resolving postpartum and depression persisting to postpartum [7]. Yet, there is lack of knowledge about psychobiological factors influencing individual susceptibility to PPD. Lately, maternal telomere length (TL) has been assessed as a potential biological marker of PPD, yet findings on maternal TL from whole blood or peripheral blood mononuclear cells and PPD are sparse [8–10], and the directionality of any association remains unclear.

Telomeres are repetitive nucleotide sequences at the ends of chromosomes that protect the chromosomes from genome instability-promoting events, such as degradation, fusion or inappropriate recombination [11]. The telomere undergoes shortening during each DNA replication cycle until it reaches a certain length, at which time cell apoptosis is signaled. Therefore, TL is often used as a biological marker for cellular senescence [12]. While being largely genetically determined, TL also depends on developmental and environmental factors [13, 14]. TL shortening is counteracted by the enzyme telomerase, a catalytic enzyme with a protein component encoded by the *Telomerase Reverse Transcriptase* (*TERT*) gene and an RNA template component encoded by *Telomerase RNA Component* (*TERC*) gene. These two components promote telomeric extension whenever a telomere becomes shortened from nuclease action and incomplete terminal DNA replication [11].

Meta-analyses have reported associations between shorter TL and psychiatric disorders [15], including depression [16], although shorter TL per se does not seem to prospectively predict higher risk of depression [16]. Moreover, polymorphisms in the *TERT* and *TERC* genes have been associated not only with TL [17, 18], but also with depression [19].

Sparse evidence is available on the relationship between mental health throughout the peripartum period and TL. *Groer* et al. reported a correlation between shorter TL, two to four months postpartum, and higher levels of anxiety postpartum, although no association was found between TL and depressive symptoms [9]. Recently, shorter TL, assessed during pregnancy, was associated with higher acculturative stress and predicted PPD in a sample of Latino mothers [8]. Additionally, low childhood socio-economic status and low current family support, but not childhood trauma, were related to shorter TL across pregnancy and postpartum [10]. Altogether, only few studies assessed the relationship between TL and depressive or anxiety symptoms peripartum, hinting towards a negative association between TL and anxiety symptoms but not PPD, but yet they did not account for genetic predisposition related to the telomere machinery.

Adverse childhood experiences (ACE) have been linked with poor physical and mental health outcomes [20, 21], including PPD [22–24]. However, the underlying mechanisms are not fully elucidated. ACE have been associated with impaired stress system response [20, 25], which may play a major role in the development of PPD [26, 27]. Mounting evidence suggests that shorter TL may be a biomarker of exposure to adversity, and not only an intrinsic feature of somatic and psychiatric disorders [28]. Indeed, ACE have been consistently associated with shorter TL [29], and could thus be an important moderator of the relationship between TL and PPD.

**Fig. 1:**
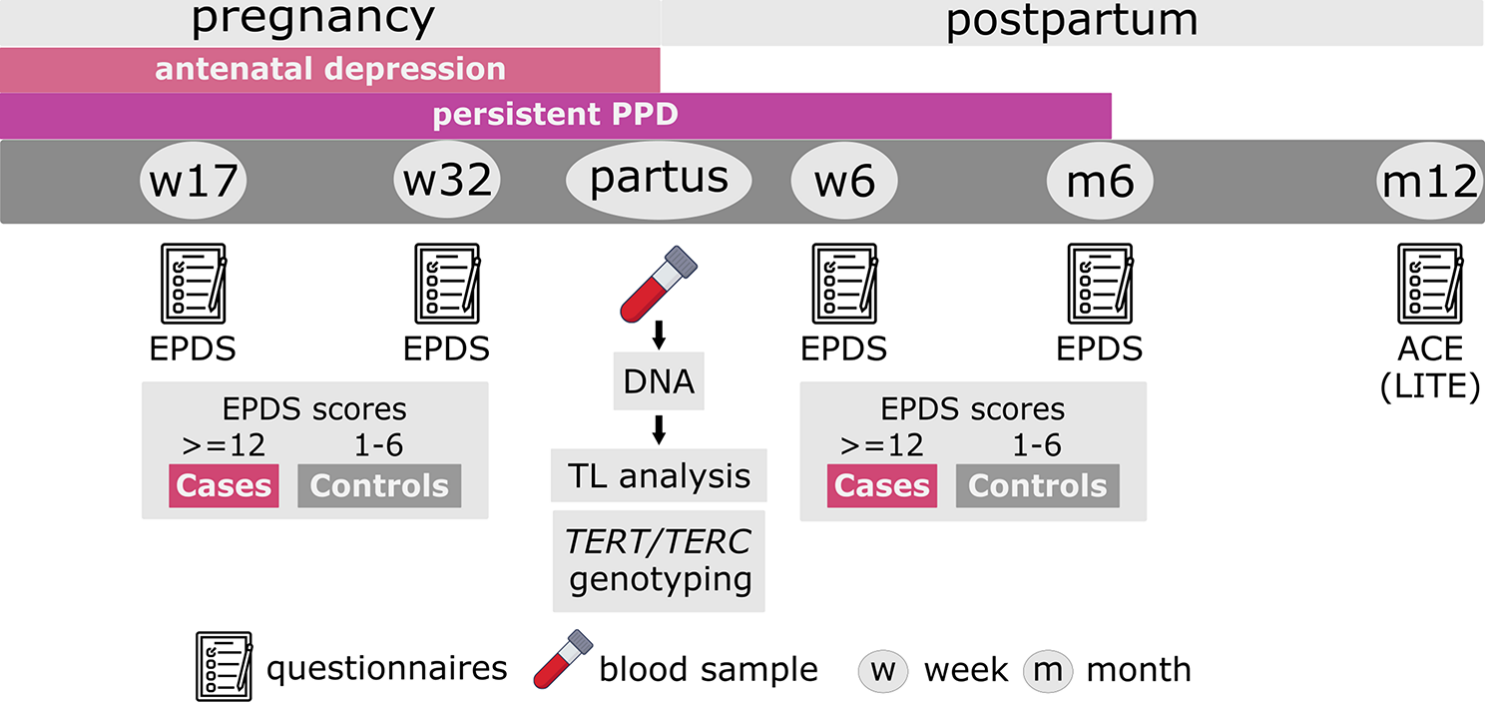
Study design. Participants self-reported their depressive symptoms using the Swedish version of the Edinburgh Postnatal Depression Scale, EPDS at weeks 17 and 32 during pregnancy, at 6-weeks and 6-months postpartum. Participants with EPDS > = 12 at any time during pregnancy only were defined as antenatal depressive symptoms (AND) group (*n* = 53); those with EPDS > = 12 at any time during pregnancy and any time during postpartum were defined as persistent PPD group (*n* = 43). Controls had EPDS < 6 throughout pregnancy and postpartum (*n* = 187). Adverse childhood experiences (ACE) up to 18 years of age were assessed using the self-report Lifetime Incidence of Traumatic Events (LITE) questionnaire at 12 months postpartum. DNA was isolated from 283 blood samples collected at delivery, and was used for Telomere Length (TL) analysis and genotyping of Telomerase Reverse Transcriptase (TERT) and Telomerase RNA component (TERC) genes

The aim of the present study was to examine the association between PPD symptoms and TL, and the possible moderating role of (i) ACE and (ii) polymorphic variations of the *TERT* and *TERC* genes involved in the telomere machinery (Fig. 1). The hypothesis was that both antenatal PPD symptoms resolving postpartum, as well as persistent PPD symptoms throughout the peripartum period would be associated with shorter TL at delivery. ACE were expected to strengthen these associations while tests on moderation effects with genotype were explorative.

## Methods

### Subjects

Candidate participants (*n* = 300) for this study were selected from the Biology, Affect, Stress, Imaging and Cognition (BASIC) cohort, a prospective study on maternal health and well-being carried out in accordance with the Declaration of Helsinki at Uppsala University Hospital, Uppsala, Sweden [30]. Participants were all pregnant, Swedish‐speaking females of ≥ 18 years of age without confidential personal data. Exclusion criteria were lifetime history of psychiatric disorders, current treatment with selective serotonin reuptake inhibitor (SSRI), and current substance use disorders. The final sample for this study was *n* = 280. The study design is illustrated in Fig. 1.

### Demographics

At pregnancy week 17, participants reported their age, education (≤ 12 vs. >12 years), employment [employed (full-time/part-time/study vs. unemployed (including sick leave/parental leave], and country of birth (foreign-born vs. Swedish‐born). Further, they retrospectively reported on height and weight before pregnancy for the calculation of body mass index (BMI), tobacco use before pregnancy (ever vs. never) and alcohol use three months before pregnancy, (more than once per week vs. once per week or less). From the delivery records, information was extracted regarding parity, pregnancy length and medical complications [7].

### Severity of depressive symptoms

Participants were asked to complete self-administered online surveys at pregnancy week 17 and 32, postpartum week 6 and month 6. Depressive symptoms were assessed with the Swedish version of the Edinburgh Postnatal Depression Scale, EPDS [31]. The EPDS has been validated for early detection of perinatal depression and recommended as a screening procedure for depressive symptoms in pregnant women [32]. Each of the 10 items on the scale is scored from 0 to 3, with total scores ranging from 0 to 30 with higher scores indicating more severe symptoms. EPDS scores of ≥ 12 during pregnancy and postpartum indicate clinically relevant depressive symptoms and were used as the clinical cut-offs for the analyses [33, 34]. Hence, participants with EPDS scores ≥ 12 at any timepoint throughout the peripartum period were assigned to the PPD symptoms group (*n* = 101), while participants with EPDS scores 1–6 during pregnancy were assigned to the control group (*n* = 199). The PPD symptoms group was further categorized into (i) participants with antenatal depressive symptoms only (antenatal depressive symptoms group (AND), *n* = 53), and (ii) participants with depressive symptoms both during pregnancy and postpartum (persistent symptoms PPD group, *n* = 43). The remaining four participants had postpartum onset PPD symptoms, but due to small sample size they were excluded from further analyses. Another 11 participants were excluded from the control group due to missing EPDS data at postpartum week six or month six. Hence, the final sample for the PPD symptoms group was *N* = 96, and the final sample for the control group was *N* = 188.

### Adverse childhood experiences (ACE)

Adversity was assessed using the Lifetime Incidence of Traumatic Events (LITE) questionnaire [35], at 12 months postpartum. LITE consists of 16 questions assessing if a traumatic event occurred, how many times it occurred, the age of the subject at the time of the first occurrence, and how much it upset the person then and now. Two indexes were created by summing the answers to items 1–8, non-interpersonal events (nIPE), including accident, death, illness or natural disaster, and to items 9–16, interpersonal events (IPE). Possible IPE were physical, emotional, or sexual acts perpetrated by another person with the intention to harm or intimidate, or without consideration of such harm [36], or witnessing others suffering such events. ACE considered only traumatic events occurring up to 18 years of age.

### Telomere length

Blood samples were collected from participants during childbirth (*n* = 284). DNA for TL analysis was isolated from the buffy coat using the silica-based Kleargene™ XL nucleic acid extraction kit (^®^LGC, UK). DNA concentration was measured by DeNovix monitoring the absorbance at 260 nm. Values above 5 ng/µl were considered acceptable. DNA purity was estimated as the ratio absorbance at 260 nm / absorbance at 280 nm. Ratios within the 1.6–2.4 range were considered acceptable. The mean TL, as represented by the ratio between telomere repeat copy number (T) and a single-copy gene [*human albumin (ALB)*], copy number (S), was determined with the quantitative polymerase chain reaction (qPCR) methodology [37]. qPCR analyses were performed using the Quant Studio™ 5 PCR System. All samples were tested in triplicates. Validation data fixed lower level at 4,700 bp and upper level at 14,400 bp with a standard deviation of 945 bp. Internal controls were included, and a regression analysis was performed for each run/plate. The plates were repeated if their regression curves had R^2^ < 0.75. The fluorescence intensities measured by qPCR were translated to bp through a standard regression curve that was generated using control cell lines of human origin, with known TL. The TL values (in bp) for the samples tested were calculated by interpolation within this standard curve. The standard regression curve’s coefficient of determination R^2^ for the plate analyzed met quality control (QC) standards (R^2^ > 0.75). All samples met the quality control thresholds regarding Coefficient of Variation (< 15%). NTC (negative control) results were within the QC standards for all plates. No amplification or amplification beyond the 37th cycle was considered not acceptable. Five cell lines have been used as control/reference. The T/S ratio for each sample was normalized to the average T/S ratio of the reference sample in order to minimize the differences between plates. TL data are presented as normalized relative T/S ratio, and bp is used as a supplementary estimate.

### Single nucleotide polymorphisms (SNP) analysis

Twenty haplotype-tagging SNPs in the *TERT* and three in the *TERC* gene were genotyped using the Illumina Global Screening Array-Multi Disease version2 (GSA-MDv2) at SciLifeLab, Uppsala, Sweden (Table S1). No-template control samples were included to enable the detection of contamination or non-specific amplification. Eleven *TERT* SNPs were excluded from further analyses: allele frequency deviated from the Hardy-Weinberg equilibrium for three *TERT* SNPs (rs10069690, rs6420020 and rs2853676), and eight *TERT* SNPs had minor allele frequency (MAF) < 0.05. For the three analyzed *TERC* SNPs, participants had the same genotype; thus, the *TERC* SNPs were also excluded from further analysis. In total, nine *TERT* SNPs were included in the final analyses (*rs2736098*,* rs2853672*,* rs2853677*,* rs2736100*,* rs7705526*,* rs2242652*,* rs2736122*,* rs7725218*,* rs56345976*). Homozygotes of the minor allele in genotypes with MAF < 30% were grouped with the heterozygous group to address the statistical constraint imposed by low MAF.

### Statistical analysis

Normality of the data was assessed using the Kolmogorov-Smirnov test. Four more samples from the controls were excluded from further analysis as outliers, based on the bps measurement which was > 20 Kbp.

Group differences in demographic variables were assessed by ANOVA, or Mann-Whitney and Kruskal-Wallis for non-normally distributed data. Chi-square test was used to assess group differences in categorical variables. Correlation analyses were performed using the non-parametric Spearman test. Key covariates included age, education level, BMI, smoking, and employment at pregnancy week 17.

Differences in TL among (i) controls (*n* = 184), (ii) participants with AND only (*n* = 53), and (iii) participants with persistent PPD (*n* = 43) were assessed with the non-parametric Kruskal-Wallis test.

Interaction effects (i.e. moderation analysis) between (i) ACE and group, (ii) *TERT* genotype and group and (iii) *TERT* genotype, ACE and group on TL were tested using the Univariate General Linear Model (GLM) test, two-way or three-way ANCOVA with Type III sum of squares, which is robust to violations of normality [38]. Differences in TL within groups between participants without ACE and those with at least on ACE were also assessed with Kruskal-Wallis test.

Correction for multiple comparisons was applied using the Bonferroni method. All analyses were carried out with SPSS 28.0.

## Results

### Demographics

The demographics of the participants (*n* = 280) are presented in Table 1. Compared with controls (*n* = 184), the groups with AND (*n* = 53) and persistent PPD (*n* = 43) symptoms were less likely to have full time employment at mid pregnancy. Females with persistent PPD symptoms had experienced more ACE compared to both controls and the AND symptom group (Table 1). Moreover, the number of ACE IPE was positively, but weakly (~ *r* = 0.2) correlated with EPDS scores throughout pregnancy and postpartum (Table S3) and individuals with PPD compared (*n* = 96) with controls (*n* = 184) reported more ACE IPE (χ² = 12.8; df = 1; *p* < 0.001).

**Table 1:** Clinical characteristics of females with depressive symptoms only during pregnancy (antenatal depression, AND), persistent depression during both pregnancy and postpartum (persistent PPD) and healthy controls (never depressed)

	AND	Persistent PPD	Controls	*P*-value
N	53	43	184	
Age (years)	30.4 ± 4.6	30.2 ± 4.4	31.5 ± 3.8	0.06
BMI (kg/m^2^)	23.9 (18.8–38.1)	24.1 (18.6–36.7)	23.1 (16.6–35.1)	0.20
Primiparity	25 (49%)	26 (58%)	101 (55%)	0.50
Education				0.1
*High school or lower*	17 (32%)	10 (23%)	34 (19%)	
*University*	36 (68%)	33 (77%)	150 (81%)	
Employment 17 pw				**0.007**
*Full time*,* part time*,* student*	47 (89%)	36 (84%)	177 (96%)	
*Parental leave*,* sick leave*,* unemployed*	6 (11%)	7 (16%)	7 (4%)	
Smoking, ever	18 (34%)	13 (30%)	44 (24%)	0.3
*Missing*	0	0	1	
EPDS, 17 pw	12 (0–19)	11 (5–18)	3 (1–6)	**< 0.001**
EPDS, 32 pw	13 (3–18)	13 (2–22)	3 (1–6)	**< 0.001**
EPDS, 6 ppw	7 (0–11)	13.5 (5–25)	3 (1–6)	**< 0.001**
*Missing*	0	1	0	
EPDS, 6 ppm	7 (0–11)	12 (3–20)	3 (0–11)	**< 0.001**
*Missing*	0	2	0	
ACE (at least 1) *IPE (at least 1)* *nIPE (at least 1)*	11 (21%) 9 (17%) 9 (17%)	20 (47%) 16 (37%) 17 (40%)	55 (30%) 18 (10%) 49 (27%)	**0.02** **< 0.001** 0.05
TL (T/S ratio) TL (bps)	1.1 (0.1–2.9) 10,163 (5921–16792)	1.1 (0.5–3.4) 9982 (7487–18898)	1.3 (0.3–3.6) 10,770 (6997–19647)	0.08

Data are presented as mean ± SD, median (min-max), or n (%). Pregnancy complications include diabetes, hypertension, and preeclampsia. BMI, body mass index; EPDS, Edinburgh Postnatal Depression Scale; pw: pregnancy week; ppw: postpartum week; ppm: postpartum month; TL: Telomere Length

### TL and PPD

**Fig. 2:**
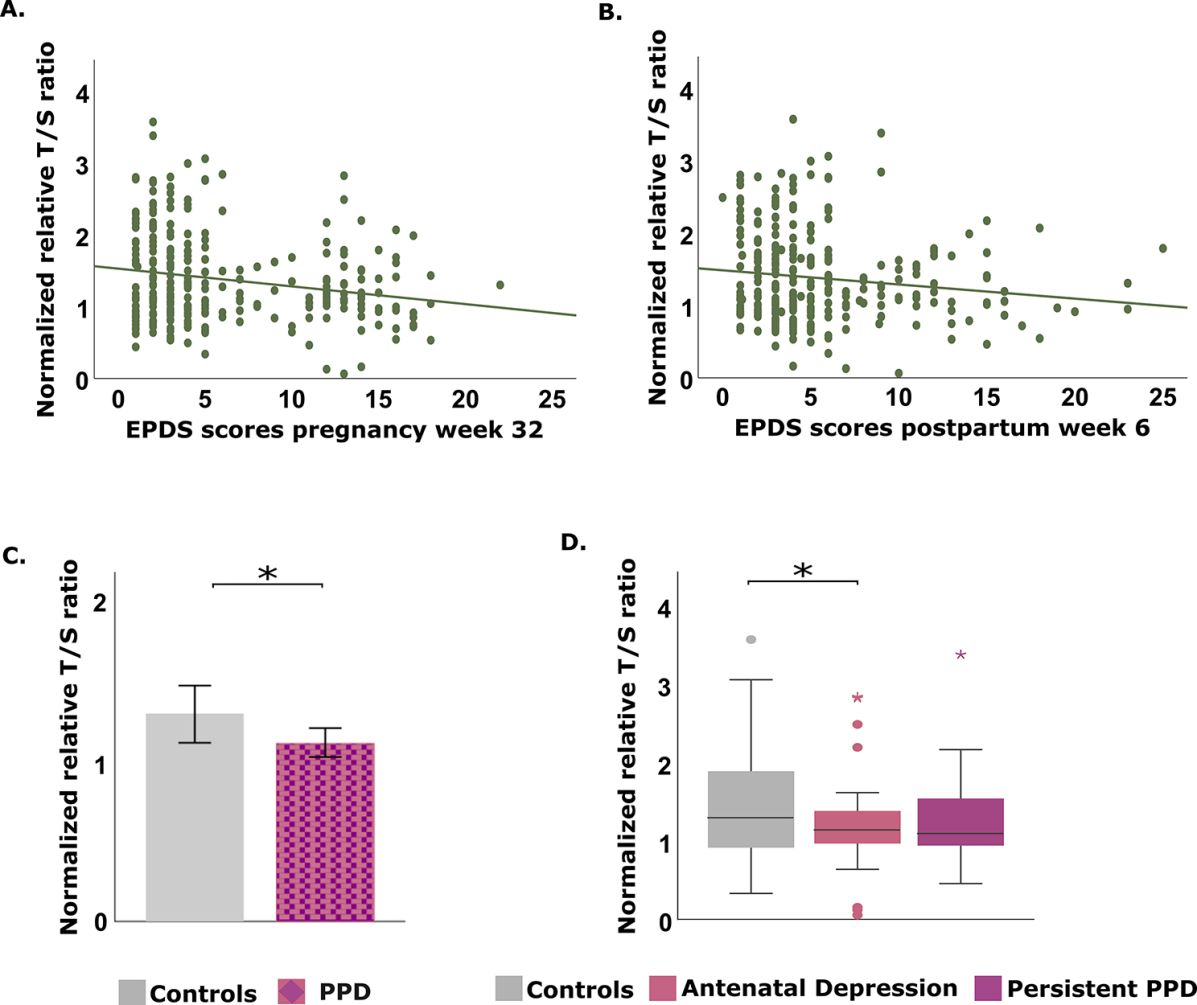
Telomere length and correlation with EPDS scores during pregnancy week 32 **(A)** and postpartum week 6 **(B)** in the whole group; **(C)** median telomere length in individuals with either antenatal or persistent PPD vs. controls; **(D)** telomere length by PPD trajectory group. AU: Arbitrary Units; EPDS: Edinburgh Postnatal Depressive Score; PPD: Peripartum Depression

In the whole sample, TL was negatively correlated with EPDS scores at 32 weeks of gestation (~ *r* = -0.2) and at 6 weeks postpartum (~ *r* = -0.1), even after controlling for potential confounders (Table 2; Fig. 2a-b). TL was significantly shorter in individuals with PPD compared (*n* = 96) with controls (*n* = 184) (*M-W U*: 7404; *p* = 0.026; Fig. 2c).

**Table 2:** Correlations between TL and severity of depressive symptoms per timepoint, unadjusted and adjusted for covariates (age, BMI, smoking, employment 17pw, education) in the whole sample and by group (controls, antenatal depression (AND) only, and persistent PPD)

	Unadjusted		Adjusted	
Whole sample (*N = 280)*	*r*	*p*	*r*	*p*
EPDS, 17 pw	-0.05	0.4	-0.07	0.04
EPDS, 32 pw	**-0.15**	**0.01**	**-0.19**	**0.002**
EPDS, 6 ppw	**-0.12**	**0.04**	**-0.12**	**0.05**
EPDS, 6 ppm	-0.11	0.07	**-0.12**	**0.05**
*Controls (* *N* * = 184)*				
EPDS, 17 pw	0.04	0.6	0.04	0.6
EPDS, 32 pw	-0.06	0.43	-0.05	0.49
EPDS, 6 ppw	-0.06	0.4	-0.04	0.56
EPDS, 6 ppm	-0.07	0.32	-0.06	0.46
*AND (* *N* * = 53)*				
EPDS, 17 pw	0.08	0.56	0.1	0.51
EPDS, 32 pw	-0.08	0.59	-0.22	0.13
EPDS, 6 ppw	0.05	0.73	-0.05	0.72
EPDS, 6 ppm	0.22	0.12	0.17	0.25
*Persistent PPD (* *N* * = 43)*				
EPDS, 17 pw	0.04	0.82	0.3	0.09
EPDS, 32 pw	-0.16	0.32	-0.32	0.06
EPDS, 6 ppw	-0.16	0.39	-0.05	0.75
EPDS, 6 ppm	-0.28	0.08	**-0.36**	**0.04**

r: Spearman’s correlation

EPDS: Edinburgh Postnatal Depression Scale; pw: pregnancy week; ppw: postpartum week; ppm: postpartum month

Regarding trajectories, AND symptoms resolving postpartum was associated with shorter TL (median = 1.1; *p* = 0.046) compared to controls (median = 1.3) (Table 1; Fig. 1d). Upon correction for multiple testing this association did not remain significant (adj. *p* = 0.14). Also, no correlation was observed between TL and EPDS scores during pregnancy in the group with AND symptoms (Table 2). TL did not differ in the subjects with persistent PPD symptoms compared to controls (Fig. 2c). However, in this group, TL was moderately and negatively correlated with EPDS scores at postpartum month 6 (*r* = -0.36) when accounting for potential confounding factors (Table 2).

### ACE, TL, and PPD

**Fig. 3:**
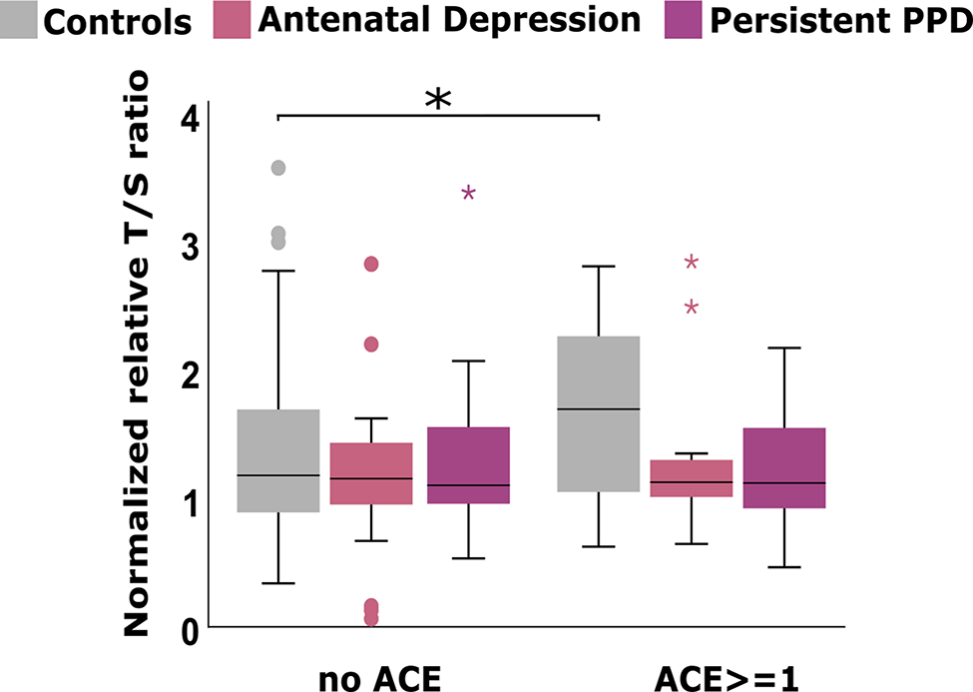
Telomere length by group in relation to adverse childhood experiences (ACE); AU: Arbitrary Units; PPD: Peripartum Depression

There was a significant interaction effect between ACE and trajectories on TL (F _(2,274)_ = 3.14; *p* = 0.045; Adj. R^2^ = 0.07; partial eta-square = 0.02), driven by shorter TL (b = -0.145, *p* = 0.017) with increasing ACE in the group with persistent PPD symptoms, whereas the opposite direction (longer TL) was observed in the controls. Among controls, TL was positively correlated with ACE (~ *r* = 0.22; *p =* 0.003) (Table S2). Additionally, control females with ACE had longer TL (median = 1.7) as compared to those without ACE (median = 1.2; *p* = 0.003) (Fig. 3), with presence of at least one IPE being the driving factor (data not shown).

### The role of SNPs in the TERT gene

Genotype frequencies of the SNPs in *TERT* gene did not differ regarding TL, or any of the covariates, i.e. maternal age, maternal BMI, education, employment, or smoking (Tables S4-12). Genotypic frequencies did not differ by trajectory group either. For SNP *rs2736098*, there was an association with EPDS score at pregnancy week 32; carriers of the minor allele (T) reported lower EPDS scores compared to CC homozygotes (Table S4). For SNP *rs56345976* there was an association with EPDS score at postpartum month 6; homozygotes of the minor allele (G) reported lower EPDS scores compared to AA/AG groups (Table S12). There was no significant interaction between the *TERT* genotype and trajectory group on TL. Lastly, there was no significant interaction between the *TERT* genotype, ACE, and trajectory group on TL.

## Discussion

The potential associations between TL and depression symptoms during pregnancy and postpartum, as well as the possible moderating role of ACE and TL-genotype, were examined in a prospective cohort. TL was negatively associated with severity of PPD symptoms during late pregnancy and early postpartum, corroborated by the shorter TL observed in individuals with PPD compared with controls.

Regarding PPD trajectories, individuals with depressive symptoms only during pregnancy, which resolved postpartum, had nominally significant shorter telomeres in compared to controls. Lastly, ACE moderated the trajectory group effect on TL. Interestingly, among subjects with depressive symptoms persisting into the postpartum period, TL was shorter with increasing ACE compared to the controls, whereas not such association was seen in the AND symptoms group. TL genotype was not associated with PPD, nor interacted with ACE to explain PPD symptom trajectories.

To date, the few studies on TL and mental health during the perinatal period have shown a negative association between the two factors. Postpartum, shorter maternal TL has been associated with higher levels of anxiety [9]. Antenatally, shorter maternal TL from whole blood has been associated with acculturative stress and predicted PPD postpartum in a sample of Latino mothers [8]. In line, we also found a negative correlation between TL and PPD symptoms severity as well as shorter maternal TL in participants with depressive symptoms compared to the controls. This suggest shorter TL as a risk factor for PPD and/or a negative impact of PPD on TL.

ACE have been consistently associated with shorter TL [29], as well as low socioeconomic status during childhood or low current family support [10]. Nevertheless, ACE were positively associated with TL among controls who had longer TL if experienced at least one ACE (IPE) compared to those without ACE. This unexpected finding could hint towards resiliency for those with longer TL, who despite exposure to ACE do not develop PPD symptoms. On the other hand, in the group with persistent PPD symptoms, who on the contrary had shorter TL with increasing ACE, suggesting a moderating role of ACE. Taken together, the present finding on ACE and TL hint towards different pathophysiology behind the PPD trajectories but may also be biased by the small size of the samples in each trajectory group who experienced ACE.

Genetic polymorphisms in the *TERT* gene have been associated with differences in mean TL [18, 39] and mental disorders. Here, lower EPDS score (about one point) was associated with the minor allele T of SNP rs2736098 and the GG genotype of SNP rs56345976. Nonetheless, none of the candidate SNPs in the *TERT* gene were associated with PPD groups, neither did they moderate the association between PPD trajectory and TL, even when ACE was considered.

The present results should be cautiously interpreted considering a number of limitations. In the present study, TL was measured in the maternal buffy coat collected at birth. Although centrifugation steps for buffy coat isolation should minimize any potential contamination of cell-free fetal DNA in the maternal blood sample, due to low molecular weight (100–180 bp) of the former [40], we cannot exclude such possibility, which might have affected the TL estimates. Furthermore, estrogen or progesterone levels, which may have affected TL [41–43], have not been measured and considered herein. Hence, the present study cannot shed light on how hormone levels during pregnancy may have affected TL, but future studies elucidating this mechanism are warranted. Though no genetic ancestry was estimated, the variable “ethnicity” based on self-reported data reflected the Caucasian origin typical of the Uppsala catchment area. Sample size of the postpartum-onset case group was too small (*N* = 4), impeding us from identifying possible associations between TL-ACE and postpartum depressive symptoms. Lastly, low statistical power due to small-size groups in PPD trajectories might explain the trend-level findings regarding their association with TL, and why a moderating role by genotype was not identified.

## Conclusions

In summary, the present findings point to a negative correlation between TL and severity of depressive symptoms at pregnancy week 32 and postpartum week 6. Additionally, PPD was associated with shorter TL. Regarding trajectories, this association was present in the group with antenatal symptoms resolving postpartum, while sample size could have hindered the identification of a similar association in the group with persistent PPD symptoms. TL-related genotype was not associated with PPD. On the other hand, a positive association between TL and ACE was found among controls. Indeed ACE, but not *TERT* genotype, moderated the relationship between PPD trajectories and TL; the group with persistent PPD symptoms had shorter TL with increasing ACE, whereas the opposite pattern (longer TL) was observed in the controls. Longer TL in the controls could potentially serve as a resilient marker, which despite exposure to ACE, might protect the individuals from developing depressive symptoms. The present findings contribute to the understanding of factors influencing mental health in vulnerable phases of a female´s life, such as pregnancy and the postpartum period.

## Electronic supplementary material

Below is the link to the electronic supplementary material.

Supplementary Material 1

## Data Availability

The datasets used and/or analyzed during the current study are available from the corresponding author upon reasonable request.

## Abbreviations

ACE: Adverse Childhood Experiences
AND: Antenatal Depression
BMI: Body Mass Index
EPDS: Edinburgh Postnatal Depression Scale
IPE: Interpersonal events
nIPE: Non-interpersonal events
LITE: Lifetime Incidence of Traumatic Events
MAF: Minor Allele Frequency
PPD: Peripartum Depression
SNP: Single Nucleotide Polymorphism
TERT: Telomerase Reverse Transcriptase
TERC: Telomerase RNA Component
TL: Telomere Length
