# Supplementary material for: Peripartum depression symptom trajectories, telomere length and genotype, and adverse childhood experiences
Source: BMC Psychiatry. 2024 Oct 8;24:661. doi: 10.1186/s12888-024-06115-1 (PMC11462957; doi:10.1186/s12888-024-06115-1)
Supplement: Supplementary file 1 — Supplementary Material 1 [file 12888_2024_6115_MOESM1_ESM.docx]

***Peripartum depression symptom trajectories, telomere length and genotype, and adverse childhood experiences***

Maria Vrettou^a^, Susanne Lager^b^, Simone Toffoletto^a^, Stavros I Iliadis^b^, Theodora Kunovac Kallak^b^, Sara Agnafors^c^, Vanessa Nieratschker^d^, Alkistis Skalkidou^b#^, Erika Comasco^a#^

1. Department of Women’s and Children’s Health, Science for Life Laboratory, Uppsala University, Uppsala, Sweden
2. Department of Women’s and Children’s Health, Uppsala University, Uppsala, Sweden
3. Department of Biomedical and Clinical Sciences, Division of Women’s and Children’s Health, Linköping University, Linköping, Sweden
4. Department of Psychiatry and Psychotherapy, Tübingen Center for Mental Health (TüCMH), Medical University Hospital Tübingen, German Center for Mental Health (DZPG), partner site Tübingen, Germany

# shared authorship

### Supplementary material

### In controls, neither age nor BMI were correlated with TL (Table S2). Age was positively correlated with TL, driven by the groups with AND and persistent PPD symptoms (Table S2). Regarding ACE, among all participants (n = 280), TL was positively, though weakly (~ r = 0.14), correlated with ACE (Table S2, Figure S1), an association driven by the controls (Table S2).

### Supplementary Tables

### Table S1. Analyzed single nucleotide polymorphisms (SNPs) in the *TERT* and *TERC* genes

| SNP | Included in analyses | Chromosomic Location |
| --- | --- | --- |
| *TERT* |  |  |
| rs2736122 | Yes | Chr5:1257506 |
| rs2242652 | Yes | Chr5:1279913 |
| rs7705526 | Yes | Chr5:1285859 |
| rs2736100 | Yes | Chr5:1286401 |
| rs2853677 | Yes | Chr5:1287079 |
| rs56345976 | Yes | Chr5:1276758 |
| rs2853672 | Yes | Chr5:1292868 |
| rs2736098 | Yes | Chr5:1293971 |
| rs7725218 | Yes | Chr5:1282299 |
| rs61748181 | No, low MAF | Chr5:1294051 |
| rs34194491 | No, low MAF | Chr5:1267098 |
| rs33948291 | No, low MAF | Chr5:1280148 |
| rs6866456 | No, low MAF | Chr5:1281578 |
| rs6554743 | No, low MAF | Chr5:1281775 |
| rs35838177 | No, low MAF | Chr5:1287175 |
| rs71595003 | No, low MAF | Chr5:1292003 |
| rs114616103 | No, low MAF | Chr5:1292843 |
| rs6420020 | No, not in HWE | Chr5:1283931 |
| rs2853676 | No, not in HWE | Chr5:1288432 |
| rs10069690 | No, not in HWE | Chr5:1279675 |
| *TERC*  rs199422287  rs141686314  rs113487931 | No, same genotype across participants  No, same genotype across participants No, same genotype across participants | Chr3:169764611  Chr3:169764833  chr3:169765003 |

HWE: Hardy Weinberg Equilibrium, MAF: minor allele frequency, SNP: single nucleotide polymorphism, TERT: telomerase reverse transcriptase gene, TERC: telomerase RNA component gene

Chromosomic location is based on GRCh38 assembly

### Table S2. Correlations between TL and ACE, BMI and age unadjusted and adjusted for covariates (age, BMI, smoking, employment 17wGa, education) for the whole sample and by group

|  | Unadjusted | | Adjusted | |
| --- | --- | --- | --- | --- |
| Whole sample | *r* | *p* | *r* | *p* |
| ACE (total)  *nIPE*  *IPE* | **0.14**  **0.15**  0.05 | **0.02**  **0.01**  0.45 | **0.15**  **0.16**  0.08 | **0.01**  **0.007**  0.18 |
| BMI (kg/m^2^) | 0.05 | 0.43 | n.a | n.a |
| Age (years) | **0.16** | **0.01** | n.a | n.a |
| *Controls (N=184)* |  |  |  |  |
| ACE (total)  *nIPE*  *IPE* | **0.22**  **0.21**  0.14 | **0.003**  **0.005**  0.06 | **0.21**  **0.18**  **0.15** | **0.005**  **0.02**  **0.04** |
| BMI (kg/m^2^) | 0.06 | 0.46 | n.a. | n.a. |
| Age (years) | 0.05 | 0.53 | n.a. | n.a. |
| *AND (N=53)* |  |  |  |  |
| ACE (total)  *nIPE*  *IPE* | 0.03  0.02  0.05 | 0.84  0.92  0.71 | **0.33**  0.28  **0.38** | **0.02**  0.06  **0.01** |
| BMI (kg/m^2^) | -0.03 | 0.83 | n.a. | n.a. |
| Age (years) | **0.31** | **0.02** | n.a. | n.a. |
| *Persistent PPD (N=43)* |  |  |  |  |
| ACE (total)  *nIPE*  *IPE* | -0.07  0.01  -0.13 | 0.67  0.37  0.40 | -0.05  -0.08  -0.01 | 0.77  0.62  0.97 |
| BMI (kg/m^2^) | -0.03 | 0.85 | n.a. | n.a. |
| Age (years) | **0.32** | **0.04** | n.a. | n.a. |

*Spearman’s correlation.*

ACE: Adverse Childhood Experiences, (n)IPE: (non) Interpersonal Events; BMI, body mass index, n.a. = not assessable

### Table S3. Correlations between adverse interpersonal events (IPE) and severity of depressive symptoms per timepoint, unadjusted and adjusted for covariates (age, BMI, smoking, employment 17pw, education) in the whole sample. There were no correlations between ACE total and EPDS

|  | Unadjusted | | Adjusted | |
| --- | --- | --- | --- | --- |
|  | *r* | *p* | *r* | *p* |
| EPDS, 17 pw | 0.21 | **<0.001** | 0.16 | **0.009** |
| EPDS, 32 pw | 0.16 | **0.007** | 0.13 | **0.03** |
| EPDS, 6 ppw | 0.17 | **0.004** | 0.19 | **0.001** |
| EPDS, 6 ppm | 0.18 | **0.002** | 0.20 | **<0.001** |

*Spearman’s correlation.*

EPDS, Edinburgh Postnatal Depression Scale; pw: pregnancy week; ppw: postpartum week; ppm: postpartum month

### Table S4. Clinical characteristics and *TERT* SNP: rs2736098

|  | CC | T | P-value |
| --- | --- | --- | --- |
| N | 149 | 119 |  |
| Age (years) | 31.3 ± 4.2 | 30.8 ± 4 | 0.37 |
| BMI (kg/m^2^) | 22.4 (18.6-37.6) | 22.6 (16.6-38.1) | 0.46 |
| *Missing* | 1 | 0 |  |
| Primiparity | 71 (49.3%) | 78 (66.7%) | **0.01** |
| *Missing* | 5 | 2 |  |
| Education |  |  | 0.16 |
| *High school or lower* | 37 (24.8%) | 21 (17.6%) |  |
| *University* | 112 (75.2%) | 98 (82.4%) |  |
| Employment 17 pw |  |  | 0.63 |
| *Full time, part time, student* | 138 (92.6%) | 112 (94.1%) |  |
| *Parental leave, sick leave, unemployed* | 11 (7.4%) | 7 (5.9%) |  |
| Smoking, ever | 42 (28.2%) | 31 (26.3%) | 0.73 |
| *Missing* | 0 | 1 |  |
| EPDS, 17 pw | 5 (0-19) | 4 (0-18) | 0.04 |
| EPDS, 32 pw | 4 (1-22) | 3 (1-18) | **0.03** |
| EPDS, 6 ppw | 4 (1-23) | 4 (1-25) | 0.82 |
| *Missing* | 0 | 1 |  |
| EPDS, 6 ppm | 4 (0-17) | 4 (0-20) | 0.35 |
| *Missing* | 2 | 0 |  |
| TL (T/S ratio)  TL (bps) | 1.3 (0.2-3.1)  10600 (6307-17644) | 1.1 (0.1-3.6)  10123 (5921-19647) | 0.55 |

Data are presented as mean ± SD, median (min-max), or n (%). Pregnancy complications include diabetes, hypertension, and preeclampsia. BMI: body mass index; EPDS: Edinburgh Postnatal Depression Scale; pw: pregnancy week; ppw: postpartum week; ppm: postpartum month; TL: Telomere Length

### Table S5. Clinical characteristics and *TERT* SNP: rs2853672

|  | AA | AC | CC | P-value |
| --- | --- | --- | --- | --- |
| N | 79 | 123 | 67 |  |
| Age (years) | 31.5 ± 4.1 | 31 ± 4.4 | 30.7 ± 3.6 | 0.50 |
| BMI (kg/m^2^) | 22.1 (18.7–37.6) | 22.2 (16.6-36.7) | 23.1 (18.7-38.1) | 0.11 |
| *Missing* | 0 | 1 | 0 |  |
| Primiparity | 37 (48.1%) | 78 (64.5%) | 35 (55.7%) | 0.07 |
| *Missing* | 2 | 2 | 3 |  |
| Education |  |  |  | 0.55 |
| *High school or lower* | 20 (25.3%) | 26 (21.1%) | 12 (17.9%) |  |
| *University* | 59 (74.7%) | 97 (78.9%) | 55 (82.1%) |  |
| Employment 17 pw |  |  |  | 0.36 |
| *Full time, part time, student* | 75 (94.9%) | 116 (94.3%) | 60 (89.6%) |  |
| *Parental leave, sick leave, unemployed* | 4 (5.1%) | 7 (5.7%) | 7 (10.4%) |  |
| Smoking, ever | 23 (29.1%) | 33 (27%) | 17 (25.4%) | 0.88 |
| *Missing* | 0 | 1 | 0 |  |
| EPDS, 17 pw | 5 (0-19) | 4 (1-18) | 4 (0-17) | 0.44 |
| EPDS, 32 pw | 4 (1-17) | 4 (1-22) | 4 (1-18) | 0.56 |
| EPDS, 6 ppw | 5 (1-16) | 4 (1-25) | 4 (1-23) | 0.24 |
| *Missing* | 0 | 1 | 0 |  |
| EPDS, 6 ppm | 4 (0-17) | 4 (0-20) | 4 (0-17) | 0.57 |
| *Missing* | 1 | 1 | 0 |  |
| TL (T/S ratio)  TL (bps) | 1.2 (0.5-2.9)  10480 (7608-16792) | 1.2 (0.1-3.6)  10509 (6176-19647) | 1.1 (0.1-2.8)  9991 (5921-16720) | 0.82 |

Data are presented as mean ± SD, median (min-max), or n (%). Pregnancy complications include diabetes, hypertension, and preeclampsia. BMI: body mass index; EPDS: Edinburgh Postnatal Depression Scale; pw: pregnancy week; ppw: postpartum week; ppm: postpartum month; TL: Telomere Length

### Table S6. Clinical characteristics and *TERT* SNP: rs2853677

|  | AA | AG | GG | P-value |
| --- | --- | --- | --- | --- |
| N | 103 | 115 | 51 |  |
| Age (years) | 31.2 ± 4.1 | 30.9 ± 4.3 | 31.3 ± 3.8 | 0.85 |
| BMI (kg/m^2^) | 22.1 (16.6-37.6) | 22.2 (18.6-38.1) | 23.2 (18.7-32.1) | 0.17 |
| *Missing* | 0 | 1 | 0 |  |
| Primiparity | 49 (48.5%) | 76 (67.9%) | 25 (51.0%) | **0.01** |
| *Missing* | 2 | 3 | 2 |  |
| Education |  |  |  | 0.91 |
| *High school or lower* | 22 (21.4%) | 26 (22.6%) | 10 (19.6%) |  |
| *University* | 81 (78.6%) | 89 (77.4%) | 41 (80.4%) |  |
| Employment 17 pw |  |  |  | 0.21 |
| *Full time, part time, student* | 96 (93.2%) | 110 (95.7%) | 45 (88.2%) |  |
| *Parental leave, sick leave, unemployed* | 7 (6.8%) | 5 (4.3%) | 6 (11.8%) |  |
| Smoking, ever | 29 (28.2%) | 30 (26.3%) | 14 (27.5%) | 0.95 |
| *Missing* | 0 | 1 | 0 |  |
| EPDS, 17 pw | 4 (0-19) | 4 (0-17) | 4 (1-17) | 0.7 |
| EPDS, 32 pw | 4 (1-18) | 4 (1-22) | 4 (1-18) | 0.97 |
| EPDS, 6 ppw | 4 (1-16) | 4 (1-25) | 4 (1-23) | 0.49 |
| *Missing* | 0 | 1 | 0 |  |
| EPDS, 6 ppm | 4 (0-18) | 4 (0-20) | 4 (0-17) | 0.72 |
| *Missing* | 1 | 1 | 0 |  |
| TL (T/S ratio)  TL (bps) | 1.2 (0.2-2.9)  10456 (6307-16792) | 1.3 (0.1-3.6)  10596 (5921-19647) | 1.1 (0.5-2.8)  9991 (7487-16720) | 0.8 |

Data are presented as mean ± SD, median (min-max), or n (%). Pregnancy complications include diabetes, hypertension, and preeclampsia. BMI: body mass index; EPDS: Edinburgh Postnatal Depression Scale; pw: pregnancy week; ppw: postpartum week; ppm: postpartum month; TL: Telomere Length

### Table S7. Clinical characteristics and *TERT* SNP: rs2736100

|  | AA | AC | CC | P-value |
| --- | --- | --- | --- | --- |
| N | 67 | 142 | 60 |  |
| Age (years) | 31.6 ± 4.2 | 31 ± 4.3 | 30.7 ± 3.6 | 0.42 |
| BMI (kg/m^2^) | 22.0 (18.6-36.7) | 23 (16.6-38.1) | 22.7 (18.7-34.7) | 0.37 |
| *Missing* | 0 | 1 | 0 |  |
| Primiparity | 31 (47.7%) | 86 (61.4%) | 33 (57.9%) | 0.18 |
| *Missing* | 2 | 2 | 3 |  |
| Education |  |  |  | 0.51 |
| *High school or lower* | 14 (20.9%) | 34 (23.9%) | 10 (16.7%) |  |
| *University* | 53 (79.1%) | 108 (76.1%) | 50 (83.3%) |  |
| Employment 17 pw |  |  |  | 0.96 |
| *Full time, part time, student* | 62 (92.5%) | 133 (93.7%) | 56 (93.3%) |  |
| *Parental leave, sick leave, unemployed* | 5 (7.5%) | 9 (6.3%) | 4 (6.7%) |  |
| Smoking, ever | 16 (23.9%) | 41 (29.1%) | 16 (26.7%) | 0.73 |
| *Missing* | 0 | 1 | 0 |  |
| EPDS, 17 pw | 5 (1-19) | 4 (0-18) | 4 (1-17) | 0.45 |
| EPDS, 32 pw | 4 (1-17) | 4 (1-22) | 3 (1-18) | 0.72 |
| EPDS, 6 ppw | 5 (1-25) | 4 (1-23) | 4 (1-23) | 0.47 |
| *Missing* | 0 | 1 | 0 |  |
| EPDS, 6 ppm | 4 (0-20) | 4 (0-18) | 3 (0-17) | 0.62 |
| *Missing* | 1 | 1 | 0 |  |
| TL (T/S ratio)  TL (bps) | 1.2 (0.5-2.6)  10295 (7608-15778) | 1.3 (0.1-3.6)  10717 (5921-19647) | 1.1 (0.5-2.8)  9871 (7487-16649) | 0.35 |

Data are presented as mean ± SD, median (min-max), or n (%). Pregnancy complications include diabetes, hypertension, and preeclampsia. BMI: body mass index; EPDS: Edinburgh Postnatal Depression Scale; pw: pregnancy week; ppw: postpartum week; ppm: postpartum month; TL: Telomere Length

### Table S8. Clinical characteristics and *TERT* SNP: rs7705526

|  | CC | A | P-value |
| --- | --- | --- | --- |
| N | 120 | 149 |  |
| Age (years) | 31.1 ± 4.1 | 31.1 ± 4.1 | 0.95 |
| BMI (kg/m^2^) | 22.4 (18.6-36.7) | 22.7 (16.6-38.1) | 0.4 |
|  | 0 | 1 |  |
| Primiparity | 66 (56.4%) | 84 (57.9%) | 0.81 |
| *Missing* | 3 | 4 |  |
| Education |  |  | 0.35 |
| *High school* | 29 (24.2%) | 29 (19.5%) |  |
| *University* | 91 (75.8%) | 120 (80.5%) |  |
| Employment 17 pw |  |  | 0.63 |
| *Full time, part time, student* | 111 (92.5%) | 140 (94%) |  |
| *Parental leave, sick leave, unemployed* | 9 (7.5%) | 9 (6%) |  |
| Smoking, ever | 33 (27.7%) | 40 (26.8%) | 0.87 |
| *Missing* | 0 | 1 |  |
| EPDS, 17 pw | 4 (1-19) | 4 (0-18) | 0.57 |
| EPDS, 32 pw | 4 (1-22) | 4 (1-18) | 0.93 |
| EPDS, 6 ppw | 4 (1-25) | 4 (1-23) | 0.9 |
| *Missing* | 1 | 0 |  |
| EPDS, 6 ppm | 4 (0-20) | 4 (0-18) | 0.88 |
| *Missing* | 2 | 0 |  |
| TL (T/S ratio)  TL (bps) | 1.3 (0.2-3.6)  10640 (6307-19647) | 1.1 (0.1-3.4)  10075 (5921-18898) | 0.06 |

Data are presented as mean ± SD, median (min-max), or n (%). Pregnancy complications include diabetes, hypertension, and preeclampsia. BMI: body mass index; EPDS: Edinburgh Postnatal Depression Scale; pw: pregnancy week; ppw: postpartum week; ppm: postpartum month; TL: Telomere Length

### Table S9. Clinical characteristics and *TERT* SNP: rs2242652

|  | GG | A | P-value |
| --- | --- | --- | --- |
| N | 160 | 109 |  |
| Age (years) | 31.2 ± 4 | 30.9 ± 4.2 | 0.54 |
| BMI (kg/m^2^) | 22.6 (18.6-37.6) | 22.4 (16.6-38.1) | 0.39 |
| *Missing* | 0 | 1 |  |
| Primiparity | 91 (57.6%) | 59 (56.7%) | 0.89 |
| *Missing* | 2 | 5 |  |
| Education |  |  | 0.45 |
| *High school or lower* | 32 (20%) | 26 (23.9%) |  |
| *University* | 128 (80%) | 83 (76.1%) |  |
| Employment 17 pw |  |  | 0.88 |
| *Full time, part time, student* | 149 (93.1%) | 102 (93.6%) |  |
| *Parental leave, sick leave, unemployed* | 11 (6.9%) | 7 (6.4%) |  |
| Smoking, ever | 48 (30.2%) | 25 (22.9%) | 0.19 |
| *Missing* | 1 | 1 |  |
| EPDS, 17 pw | 4 (1-19) | 4 (0-17) | 0.34 |
| EPDS, 32 pw | 4 (1-18) | 4 (1-22) | 0.9 |
| EPDS, 6 ppw | 4 (1-25) | 4 (1-23) | 0.23 |
| *Missing* | 1 | 1 |  |
| EPDS, 6 ppm | 4 (0-20) | 3 (0-17) | 0.73 |
| *Missing* | 1 | 1 |  |
| TL (T/S ratio)  TL (bps) | 1.2 (0.1-3.6)  10538 (6176-19647) | 1.1 (0.1-3.1)  10093 (5921-17644) | 0.19 |

Data are presented as mean ± SD, median (min-max), or n (%). Pregnancy complications include diabetes, hypertension, and preeclampsia. BMI: body mass index; EPDS: Edinburgh Postnatal Depression Scale; pw: pregnancy week; ppw: postpartum week; ppm: postpartum month; TL: Telomere Length

### Table S10. Clinical characteristics and *TERT* SNP: rs2736122

|  | GG | A | P-value |
| --- | --- | --- | --- |
| N | 151 | 118 |  |
| Age (years) | 31.2 ± 4.2 | 30.9 ± 4 | 0.51 |
| BMI (kg/m^2^) | 22.7 (18.7-38.1) | 22.2 (16.6-37.6) | 0.75 |
| *Missing* | 1 | 0 |  |
| Primiparity | 84 (56.8%) | 66 (57.9%) | 0.85 |
| *Missing* | 3 | 4 |  |
| Education |  |  | 0.1 |
| *High school* | 38 (25.2%) | 20 (16.9%) |  |
| *University* | 113 (74.8%) | 98 (83.1%) |  |
| Employment 17 pw |  |  | 0.96 |
| *Full time, part time, student* | 141 (93.4%) | 110 (93.2%) |  |
| *Parental leave, sick leave, unemployed* | 10 (6.6%) | 8 (6.8%) |  |
| Smoking, ever | 43 (28.5%) | 30 (25.6%) | 0.61 |
| *Missing* | 0 | 1 |  |
| EPDS, 17 pw | 4 (0-19) | 4 (1-18) | 0.35 |
| EPDS, 32 pw | 4 (1-17) | 4 (1-22) | 0.22 |
| EPDS, 6 ppw | 4 (1-25) | 4 (1-23) | 0.8 |
| *Missing* | 1 | 0 |  |
| EPDS, 6 ppm | 3.5 (0-16) | 4 (0-20) | 0.13 |
| *Missing* | 1 | 1 |  |
| TL (T/S ratio)  TL (bps) | 1.1 (0.1-3.6)  10163 (5921-19647) | 1.3 (0.1-3.4)  10728 (6176-18898) | 0.55 |

Data are presented as mean ± SD, median (min-max), or n (%). Pregnancy complications include diabetes, hypertension, and preeclampsia. BMI: body mass index; EPDS: Edinburgh Postnatal Depression Scale; pw: pregnancy week; ppw: postpartum week; ppm: postpartum month; TL: Telomere Length

### Table S11. Clinical characteristics and *TERT* SNP: rs7725218

|  | GG | GA | AA | P-value |
| --- | --- | --- | --- | --- |
| N | 114 | 121 | 33 |  |
| Age (years) | 31.1 ± 4.1 | 31.1 ± 4.2 | 30.5 ± 3.7 | 0.67 |
| BMI (kg/m^2^) | 22.3 (18.6-36.7) | 23.1 (16.6-38.1) | 21.6 (18.7-34.7) | 0.13 |
| Primiparity | 64 (57.7%) | 67 (56.3%) | 19 (61.3%) | 0.88 |
| *Missing* | 3 | 2 | 2 |  |
| Education |  |  |  | 0.69 |
| *High school* | 27 (23.7%) | 24 (19.8%) | 6 (18.2%) |  |
| *University* | 87 (76.3%) | 97 (80.2 %) | 27 (81.8%) |  |
| Employment 17 pw |  |  |  | 0.91 |
| *Full time, part time, student* | 107 (93.9%) | 112 (92.6%) | 31 (93.9%) |  |
| *Parental leave, sick leave, unemployed* | 7 (6.1%) | 9 (7.4%) | 2 (6.1%) |  |
| Smoking, ever | 32 (28.3%) | 32 (26.4%) | 9 (27.3%) | 0.95 |
| *Missing* | 1 | 0 | 0 |  |
| EPDS, 17 pw | 4 (1-19) | 4 (0-18) | 4 (1-15) | 0.64 |
| EPDS, 32 pw | 4 (1-17) | 4 (1-22) | 4 (1-18) | 0.73 |
| EPDS, 6 ppw | 4 (1-25) | 4 (1-20) | 4 (1-23) | 0.93 |
| *Missing* | 1 | 0 | 0 |  |
| EPDS, 6 ppm | 4 (0-20) | 4 (0-18) | 4 (0-13) | 0.73 |
| *Missing* | 1 | 1 | 0 |  |
| TL (T/S ratio)  TL (bps) | 1.3 (0.5-3.6)  10704 (7608-19647) | - 1. (0.1-3.4)   10113 (5921-18898) | 1.3 (0.5-2.8)  10786 (7487-16625) | 0.22 |

Data are presented as mean ± SD, median (min-max), or n (%). Pregnancy complications include diabetes, hypertension, and preeclampsia. BMI: body mass index; EPDS: Edinburgh Postnatal Depression Scale; pw: pregnancy week; ppw: postpartum week; ppm: postpartum month; TL: Telomere Length

### Table S12. Clinical characteristics and *TERT* SNP: rs56345976

|  | AA | AG | GG | P-value |
| --- | --- | --- | --- | --- |
| N | 99 | 134 | 36 |  |
| Age (years) | 31.2 ± 4.1 | 30.9 ± 4.3 | 31.6 ± 3.6 | 0.66 |
| BMI (kg/m^2^) | 23 (18.6-34.9) | 22.6 (16.6-38.1) | 23.3 (18.9-34.7) | 0.31 |
| Primiparity | 49 (50.5%) | 82 (63.6%) | 19 (52.8%) | 0.12 |
| *Missing* | 2 | 5 | 0 |  |
| Education |  |  |  | 0.94 |
| *High school or lower* | 22 (22.2%) | 29 (21.6%) | 7 (19.4%) |  |
| *University* | 77 (77.8%) | 105 (78.4 %) | 29 (80.6%) |  |
| Employment 17 pw |  |  |  | 0.78 |
| *Full time, part time, student* | 91 (91.9%) | 126 (94%) | 34 (94.4%) |  |
| *Parental leave, sick leave, unemployed* | 8 (8.1%) | 8 (6%) | 2 (5.6%) |  |
| Smoking, ever | 24 (24.2%) | 41 (30.8%) | 8 (22.2%) | 0.41 |
| *Missing* | 0 | 1 | 0 |  |
| EPDS, 17 pw | 5 (1-17) | 4 (0-19) | 4 (1-18) | 0.07 |
| EPDS, 32 pw | 4 (1-22) | 4 (1-17) | 4 (1-17) | 0.2 |
| EPDS, 6 ppw | 4 (1-23) | 4 (1-25) | 4 (1-23) | 0.14 |
| *Missing* | 0 | 0 | 1 |  |
| EPDS, 6 ppm | 5 (0-20) | 4 (0-18) | 3 (0-14) | **0.04** |
| *Missing* | 2 | 0 | 0 |  |
| TL (T/S ratio)  TL (bps) | 1.3 (0.2-3.1)  10680 (6307-17644) | 1.1 (0.1-3.4)  10085 (5921-18898) | 1.3 (0.5-3.6)  10729 (7822-19647) | 0.3 |

Data are presented as mean ± SD, median (min-max), or n (%). Pregnancy complications include diabetes, hypertension, and preeclampsia. BMI: body mass index; EPDS: Edinburgh Postnatal Depression Scale; pw: pregnancy week; ppw: postpartum week; ppm: postpartum month; TL: Telomere Length
